# Comparative Assessments of PET and ASL MR Perfusion in the Evaluation of Early Dementia

**DOI:** 10.15388/Amed.2025.32.1.10

**Published:** 2025-02-18

**Authors:** Savyasachi Jain, Shailesh B Gaikwad, Bheru Dan Charan, Anu Gupta, Madhvi Tripathy

**Affiliations:** 1 Department of Neuroimaging & Interventional Neuroradiology, All India Institute of Medical Sciences, New Delhi, India; 2 Department of Radiology, All India Institute of Medical Sciences, Rishikesh, India; 3 Department of Neurology, All India Institute of Medical Sciences, New Delhi, India; 4 Department of Nuclear Medicine, All India Institute of Medical Sciences, New Delhi, India

**Keywords:** dementia, neurodegeneration, MRI, demencija, neurodegeneracija, MRT

## Abstract

**Background:**

Overtly morbid dementing diseases have a prodromal MCI (mild cognitive impairment) phase which is crucial to recognize. Clinical scores provide an easy bedside assessment tool for holistic cognitive evaluation but fail to provide lead time. While routine biomarkers of brain atrophy are late to appear, non-contrast MRI perfusion studies like ASL may serve as a valuable alternative to 18F-FDG-PET for the recognition and classification of the degree of neurodegeneration in individuals with MCI, especially when FDG-PET is not available. Our study adds confidence as we noted brain regions where PET-ASL concordance was most robust and devised concordance with ACE-3 scores.

**Material and methods:**

We conducted a prospective study from Jan 2021 to Jan 2024. Cases were selected based on the inclusion and exclusion criteria, which have objective cognitive impairment. Healthy controls were selected. MRI and PET scans were performed in all cases. Perfusion values of arterial spine labeling, PET, and clinical examination were recorded.

**Results:**

We included 33 patients and 15 healthy controls in the study. We compared ASL and PET for all selected individuals. Our study showed that ASL can detect a hypo-perfusion region with a 91% sensitivity, 85.98% specificity, 89.8% PPV, and 87.62% NPV, with a diagnostic accuracy of 88.9%.

**Conclusion:**

ASL was a dependable replacement for the gold-standard FDG-PET. ASL may serve as a valuable alternative to ^18^F-FDG-PET for classifying the degree of neurodegeneration in individuals with prodromal AD, especially when FDG-PET is unavailable.

## Abstract (it)

**Įvadas:**

Labai svarbu atpažinti atvirų demencijos ligų prodrominę MCI (lengvo pažinimo sutrikimo) fazę. Klinikiniai balai yra paprasta prie lovos esanti holistinio kognityvinio vertinimo priemonė, tačiau jie nesuteikia galimybės numatyti trukmės. Nors įprastiniai smegenų atrofijos biologiniai žymenys pasirodo pavėluotai, nekontrastiniai MRT perfuzijos tyrimai, pavyzdžiui, ASL, gali būti vertinga 18F-FDG-PET alternatyva atpažįstant ir klasifikuojant neurodegeneracijos laipsnį asmenų, sergančių MCI, ypač kai nėra galimybės atlikti FDG-PET. Mūsų tyrimas suteikia pasitikėjimo, nes atkreipėme dėmesį į smegenų sritis, kuriose PET ir ASL suderinamumas buvo patikimiausias, ir nustatėme suderinamumą su ACE-3 balais.

**Metodai:**

Atlikome perspektyvųjį tyrimą (nuo 2021 m. sausio iki 2024 m. sausio). Pagal pacientų įtraukimo ir neįtraukimo į tyrimą kriterijus buvo atrinkti atvejai, kai nustatytas objektyvus pažinimo sutrikimas. Buvo atrinkta sveikų pacientų kontrolinė grupė. Visiems tiriamiesiems buvo atlikti magnetinio rezonanso ir PET tyrimai. Registruotos arterinio stuburo žymėjimo, PET ir klinikinio tyrimo perfuzijos vertės.

**Rezultatai:**

Tyrime dalyvavo 33 pacientai ir 15 sveikų kontrolinių asmenų. Palyginome visų atrinktų asmenų ASL ir PET. Mūsų tyrimas parodė, kad ASL gali aptikti hipoperfuzijos sritį, kurios jautrumas 91 %, specifiškumas 85,98 %, PPV 89,8 % ir NPV 87,62 %, o diagnostinis tikslumas 88,9 %.

**Išvada:**

ASL buvo patikimas auksinio standarto FDG-PET pakaitalas. ASL gali būti vertinga 18F-FDG-PET alternatyva, klasifikuojant neurodegeneracijos laipsnį asmenų, sergančių prodromine Alzheimerio liga, ypač kai FDG-PET negalima atlikti.

## Introduction

Neurodegenerative diseases leading to dementia significantly impact *disability-adjusted life-years* (DALY) [1]. Dementia prevalence has been rapidly rising, with projections indicating 131.5 million affected individuals by 2050 [2]. Managing primary neurodegenerative disorders, such as tauopathies and synucleinopathies, remains challenging due to limited treatment options [3]. *Mild Cognitive Impairment* (MCI) [4] lasts for decades before resulting in overt dementia, and it must be recognized in its nascent stage to prevent patient morbidities later [5]. ACE-3 [6,7] is a straightforward measure of global cognition for assessing five cognitive domains: attention, memory, language, verbal fluency, and visuospatial abilities. Also, depending on the period of education, MCI is defined as ACE-3 <89 (duration of education >10 years), while <86 (education duration <10 years). A cut-off score of 61 differentiates mild from moderate dementia [8]. Imaging techniques that assess regional physiological parameters, such as perfusion or glucose metabolism enhance sensitivity and allow for an earlier diagnosis in diseases with a significant clinical-pathological overlap [9]. ^18^F-Fluorodeoxyglucose Positron Emission Tomography (^18^F-FDG-PET) is the most reliable functional biomarker for neurodegeneration [10–13]. It is denoted by a high sensitivity and predictive value in detecting metabolic changes during the preclinical and prodromal stages of Alzheimer’s disease, known as *Mild Cognitive Impairment* (MCI) [8,11,14]. *Arterial spin labeling* (ASL) has been suggested as a biomarker for assessing the physiological state of the brain in patients with a suspected neurodegenerative disease [15,16]. *Arterial Spin Labeling sequence in MRI* (ASL-MRI) quantitatively measures the regional *cerebral blood flow* (CBF). It represents a potentially appealing functional imaging modality for diagnosing and monitoring neurodegenerative diseases like AD [17]. ASL cannot require exogenous contrast or radiation exposure and can be included in patients’ routine diagnostic imaging work-ups. Regional CBF and glucose metabolism are generally tightly coupled as synaptic activity is proportional to it [18]. Thus, regional ASL-CBF measures are expected to closely parallel metabolic changes on FDG-PET. Researchers have noted an overlap between areas of hypoperfusion measured with ASL-MRI and hypometabolism measured by 18F-FDG-PET acquired on the same day in mild-to-moderate AD patients [19]. Our study attempts to add to the existing literature on an early prediction of cognitive abnormalities by using these novel techniques. Our study aims to use MRI techniques and sequences in cognitively poor patients to prove the usefulness of non-radiation modality over FDG-PET, which can be used for specialized biomarker studies if required. We have compared and correlated the diagnostic value of non-contrast perfusion MR-ASL with invasive FDG-PET in patients with cognitive impairment.

## Material and methods

We conducted a prospective study at the Department of Neuroimaging and Interventional Radiology, and at the Department of Nuclear Medicine, AIIMS, New Delhi, India, from January 2021 to January 2024. All the prospective patients and controls provided the appropriate informed consent before being included in the study. The clinical history was investigated, and neurological examination was performed before implementing the imaging. Specifically, a senior neurologist with more than 10 years of experience in dementia clinical examination performed the cognitive assessment before imaging. Imaging was reviewed by two neuroradiologists and a nuclear medicine consultant. Our controls were patients who had been enrolled for MRI tests for other non-cognitive complaints like headache and were found to possess a normal MRI on base sequences (FLAIR, T1, T2, DWI, SWI, and ASL). Our controls did not undergo a PET scan.

### Inclusion Criteria

- Age between 50 and 90;
- Objective cognitive impairment (ACE 3 score <89);
- Etiologically: AD or non-AD cause of cognitive impairment.

### Exclusion Criteria

- MRI contraindicated, or the patient was uncooperative;
- History of Axis I Psychiatric disease or substance abuse (19);
- Objective evidence or clinical history of secondary causes of dementia, like:
  - Significant subcortical ischemic changes, Fazekas scale for WM lesion ≥1 for subjects ≤70 years old; Fazekas Score ≥2 for subjects >70 years old; Lacunar infarct, diameter >2 cm, number of lesions ≥2 (20);
  - Strategic infarcts;
  - Structural abnormalities in the brain;
  - Extra-axial hemorrhages;
  - Normal Pressure Hydrocephalus.

#### Imaging acquisition

All patients underwent MRI and FDG-PET within 15 days of each other.

##### PET-CT Acquisition

PET/CT was performed approximately at 60 minutes after intravenous injection of 222-296 MBq (6–8 mCi) of F-18 FDG. Imaging was performed on a *Siemens Biograph* mCT. The initial scout of the head was followed by non-contrast CT acquisition (110 mA, 120 kVp) for attenuation correction and anatomical co-registration. This was followed by a single bed, 3-D PET emission scan for 10 minutes. PET images were reconstructed (ultra HD-PET) by iterative reconstruction (5 iterations and 21 subsets). The FDG images of each case were further analyzed by using *Cortex ID* (*GE Healthcare*, Waukesha, WI) on an *ADW 4.7* (*GE Healthcare*) workstation. The *Cortex ID* application involves the generation of brain maps compared with a commercially available comprehensive database of regular brain scans and normalized globally (or to the pons/cerebellum). Automated voxel-by-voxel Z-scores were generated for the cortical *region of interest* (ROI), which included parietal, frontal, lateral temporal, mesial temporal, pre-cuneus, cingulate (anterior and posterior), and occipital cortices. Z-score = [mean subject-mean database]/standard deviation database. The voxel-based color-coded statistical analysis of the average Z-scores displayed the magnitude of each region’s metabolic change. A Z-score threshold of 2.5, corresponding to a p-value of 0.05 (two-tailed), was applied to demarcate significant abnormalities, with negative Z-scores indicating hypometabolism.

##### MRI Acquisition

The MRI scan was acquired by employing the *3T Philips Machine* (®*Ingenia Edition*) in the NMR and MRI facility of AIIMS, New Delhi, while using the 32-channel head coil. The standard dementia protocol and special sequences were acquired as follows:

- Sag-3D-T1;
- Sag-3D-FLAIR;
- Sag-3D-T2;
- SWI;
- ASL.

##### ASL

ASL acquisition was performed on a *1.5 T GE MRI Machine*. ASL perfusion imaging was performed by using the 3D pseudo-continuous labeling technique under the following parameters: TR=5613 ms, TE=10.7 ms, NEX=3, spiral read out of 8 arms x 512 samples, FOV=240X40mm, duration: 5 min 26 seconds. The post-labeling delay at our institute is age-tailored. In children between 1 to 10 years, the delay was 1025 and 1525; in the 10–30 years age group, it was 1525 and 2025. In the 30 to 60 years age group, it was 2025 and 2525, and, when the patients are aged more than 60 years, it was 2525 and 3025 ms.

PET and ASL were simultaneously seen for apparent visual concordance. Further, GE processing software ®*ReadyView* was used for ASL processing. ROI was drawn manually in regions with significant hypo-perfusion on PET (Z <-2.5) and in </=5 areas with maximum perfusion (Z > 1). The rCBF values were given as a percentage of pons rCBV to get an internal control. The mean rCBF was derived from all areas, and hypo-perfusion/hyper-perfusion was concluded based on mean rCBF.

#### Statistical Analysis

The categorical variables were presented as numbers and percentages (%). On the other hand, the quantitative data were presented as the means ± SD and the median with the 25^th^ and 75^th^ percentiles (in the interquartile range). The data normality was checked by using the Shapiro-Wilk test. We used nonparametric tests for cases in which the data was not standard. The following statistical tests were applied to the results:

1. The comparison of the quantitative and not normally distributed variables in nature was analyzed by using the Mann-Whitney test (for two groups), and quantitative and generally distributed variables in nature were analyzed by using the independent t-test. The paired t-test was used for comparison.
2. The Spearman rank correlation coefficient was used for the correlation of ACE-3 score with differences of the highest and lowest PET values.
3. The sensitivity, specificity, positive, and negative predictive values of the ASL technique were calculated to predict hypoperfusion, when taking FDG-PET hypometabolism as standard.
4. The receiver operating characteristic curve was used to assess the cut-off point of the ACE-3 score for predicting concordance of the ASL and PET Z score (-).

The data entry was done in the *Microsoft Excel* spreadsheet, and the final analysis was conducted by using the *Statistical Package for Social Sciences* (SPSS) software developed by the *IBM* manufacturer, Chicago, USA, version 25.0. For statistical significance, a p-value of less than 0.05 was considered statistically significant.

## Results

We enrolled 33 patients (variable ACE-3 scores) and 15 willing controls. We compared the ASL and PET values for all the selected individuals. In our study, most patients recruited were between the ages of 42 and 78. 39.3% of our patients were between 42 and 54, 30.3% between 54 and 66, and 27.2% between 66 and 78. Only one patient was 90 years old. The mean age of the patients was 59.3. In the study, 37.04% of the cases had an ACE-3 score ranging from 51 to 75 and >75 in each instance. Additionally, 18.52% of the cases scored between 1–25, while 7.41% scored between 26–50. The mean ACE-3 score among the study subjects was 60.93 ± 27.2. The median (25^th^–75^th^ percentile) was 70 (50–82.5). Our study included an approximately equal number of MCI and dementia patients, 48.4% (16) and 51.6% (17), respectively. The parietal lobe was the most common location of atrophy (see Table 1).

**Figure 1A:**
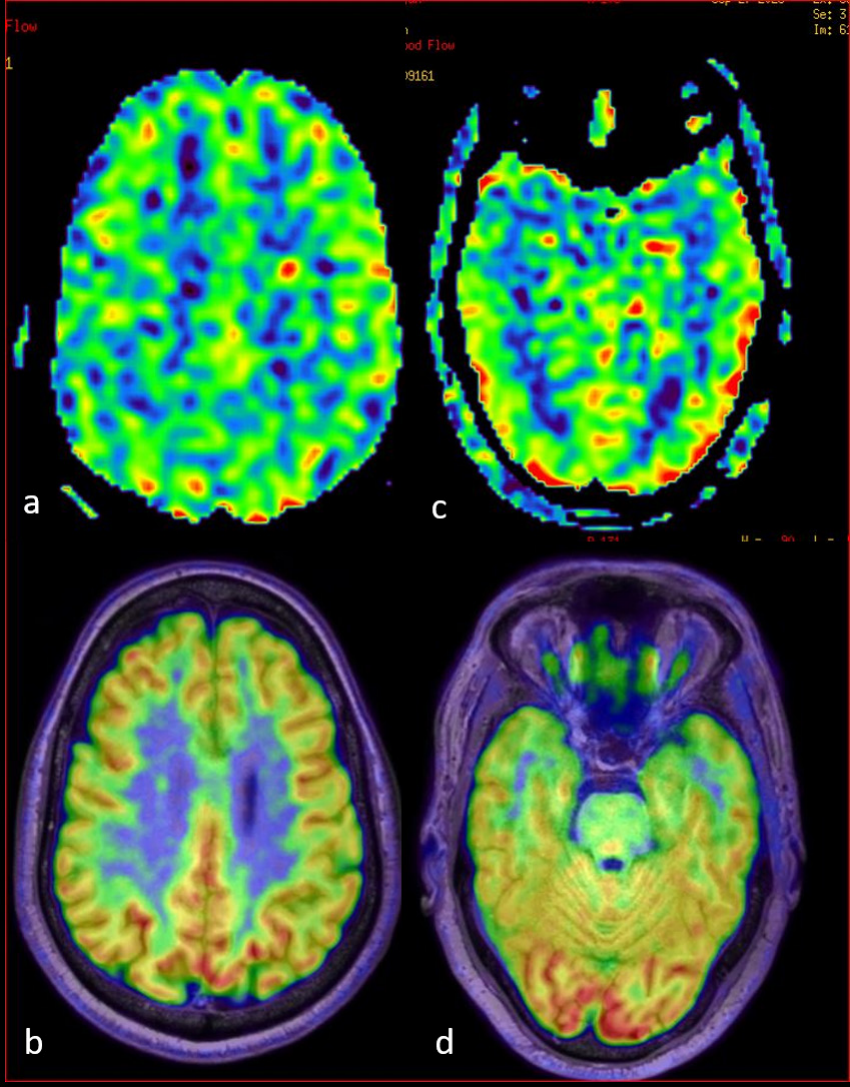
A 61-year-old with an ACE-3 score of 92 shows gross visual concordance between ASL (a, c) and PET (b, d) in areas of significant FDG-PET hypo-metabolism. ASL (a) and the corresponding PET-MR fusion image (b) show hypo-perfusion in the bilateral posterior frontal region (approx. Z score -4.2 on FDG-PET). ASL (c) and the corresponding PET (d) show hypo-perfusion in the bilateral anterior temporal region (approx. Z Score -2.2 on FDG-PET)

**Figure 1B:**
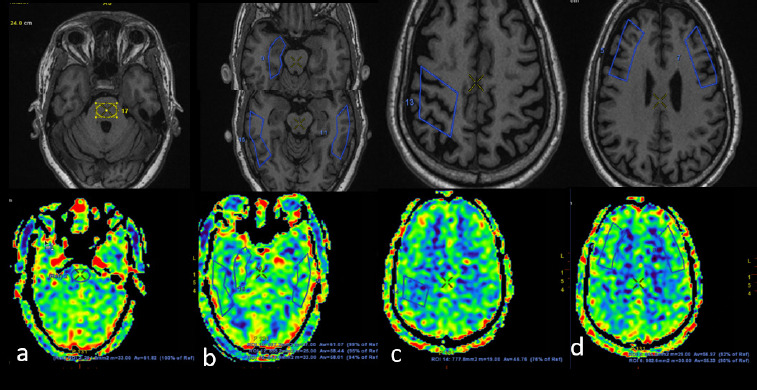
In the same patient, manual ROI was drawn on the ASL image (lower row), which showed significant FDG-PET hypometabolism, taking pons (a) as a reference. (The upper row shows a T1W image of an anatomical landmark.) ROIs and their relative percentage of perfusion are shown for bilateral temporal and right mesial temporal region (b), correct posterior frontal (c), and bilateral lateral frontal region (d)

**Figure 2A:**
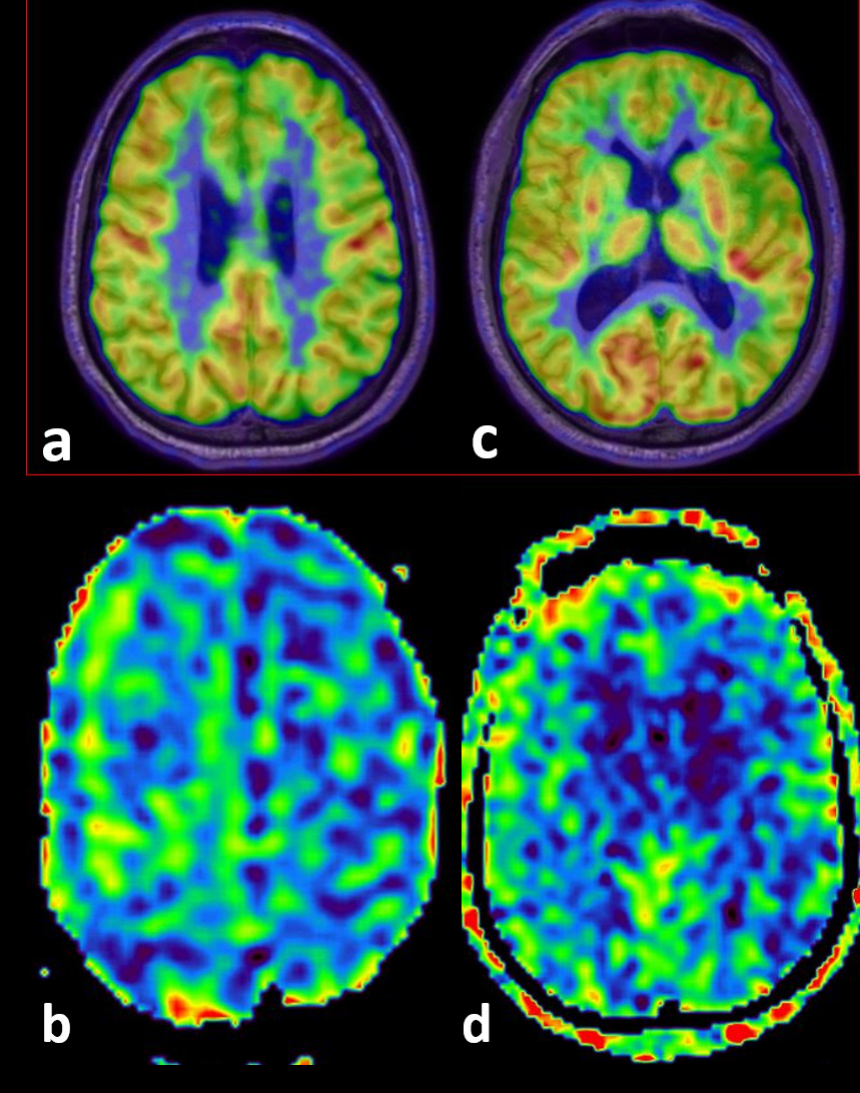
A 53-year-old with an ACE-3 score of 81 shows gross visual concordance between ASL (b, d) and PET (a, b) in areas of significant FDG-PET hypo-metabolism. The corresponding PET and ASL images (a, b) show hypo-perfusion in the left frontal region (approx. Z Score -7.5 on FDG-PET), and images (c, d) show hypo-perfusion in the left parietal region (approx. Z Score -5.5 on FDG-PET)

**Figure 2B:**
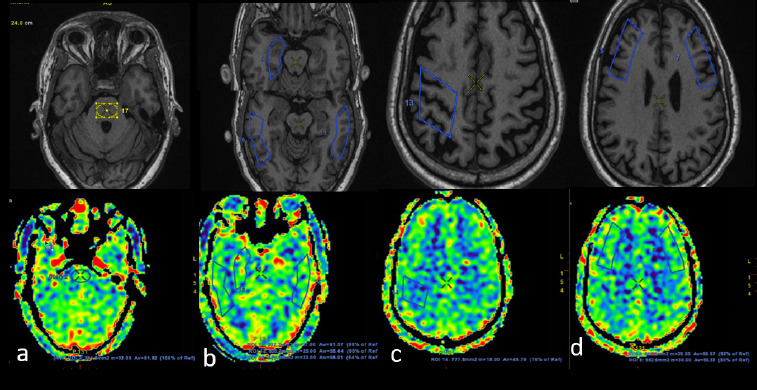
In the same patient, manual ROI was drawn on the ASL image (lower row), which showed significant FDG-PET hypometabolism, taking pons (a) as a reference (upper row shows T1W image for an anatomical landmark). ROIs and their relative percentage of perfusion are shown for the left occipital (b), bilateral lateral frontal, left parietal (c), and the left medial frontal region

**Figure 3A:**
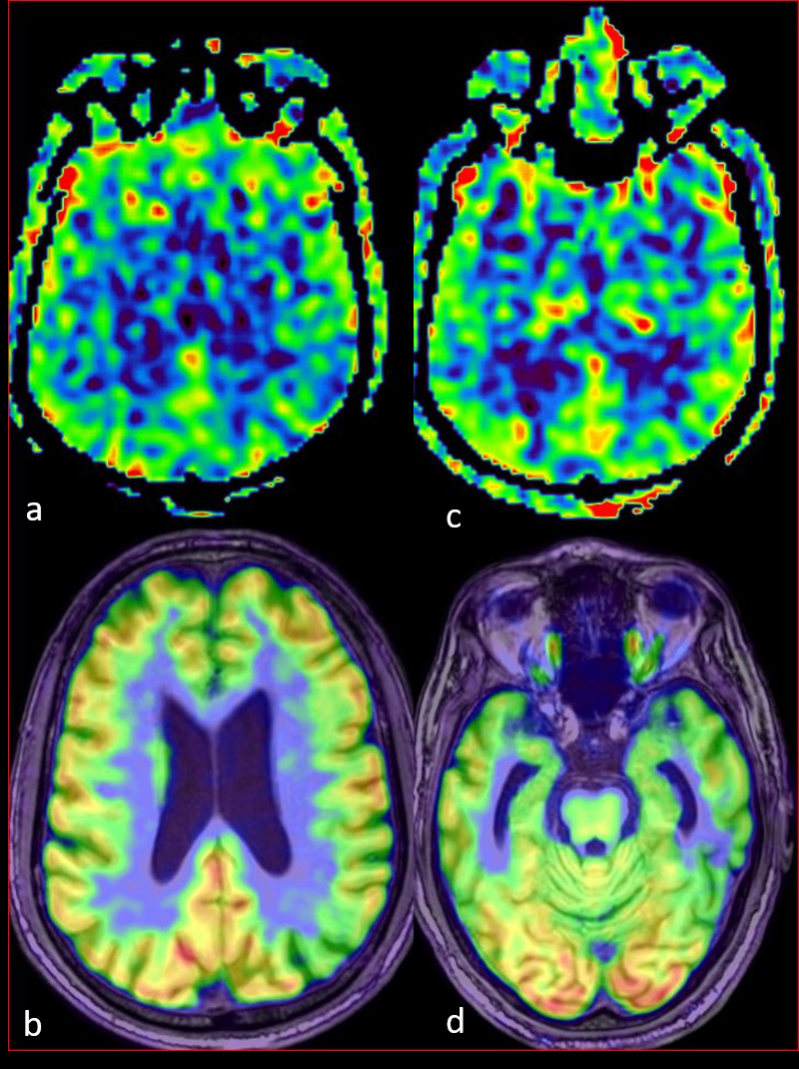
A 66-year-old with an ACE-3 score of 67 shows gross visual concordance between ASL and PET in areas of significant FDG-PET hypo-metabolism. The corresponding ASL (a) and PET (b) images show hypo-perfusion in the left parietal region (approx. Z Score -6 on FDG-PET), and other ASL (c) and PET (d) images show hypo-perfusion in left>right lateral temporal region (approx. Z Score -4 on FDG-PET)

**Figure 3B:**
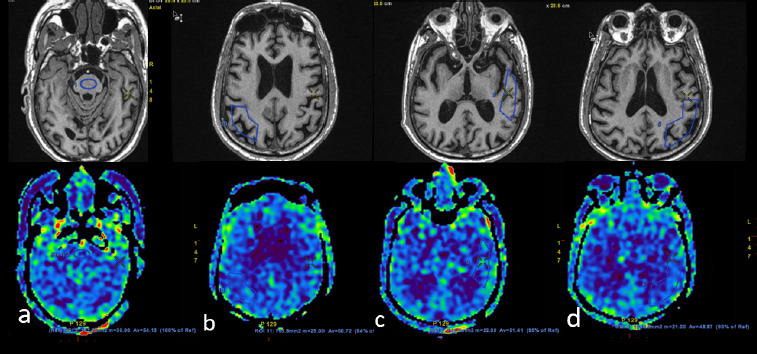
In the same patient, manual ROI was drawn on the ASL image (lower row), which shows significant FDG-PET hypometabolism, taking pons (a) as a reference (upper row shows T1W image for an anatomical landmark). ROIs and their relative percentage of perfusion are shown in the right parietal (b), left lateral temporal (c), and left lateral temporal region

**Table 1:** Predominant atrophy distribution

Grade 1 atrophy	Frequency	Percentage
Frontal	7	21.21%
Temporal	17	51.52%
Parietal	29	87.88%
Occipital	2	6.06%

The study subjects exhibited a mean PET Z score of -5.73 ± 2.9, with a median (25^th^–75^th^ percentile) of -4.9 (-3.4 to -7.2). A significant moderately negative correlation was seen between the ACE-3 score with a difference of the highest and lowest PET values with a correlation coefficient of -0.453.

It clearly shows that as the ACE-3 clinical score of the patient increases, whereas the brain shows a more uniform metabolism with a reduced range of PET Z score values.

**Table 2:** Sensitivity, specificity, positive predictive value, and negative predictive value of ASL-PET concordance

Variables	Values
Sensitivity (95% CI)	91.03% (85.16% to 95.14%)
Specificity (95% CI)	85.98% (77.93% to 91.94%)
AUC (95% CI)	0.89 (0.84 to 0.92)
Positive Predictive Value (95% CI)	89.80% (83.73% to 94.18%)
Negative Predictive Value (95% CI)	87.62% (79.76% to 93.24%)
Diagnostic accuracy	88.89%

**Chart 1:**
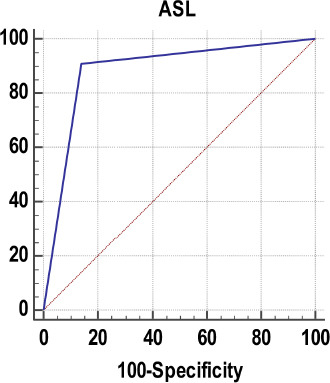
Receiver operating characteristic curve of ASL for predicting hypoperfusion

Table 2 presents the diagnostic performance of *Arterial Spin Labeling* (ASL) for predicting hypoperfusion. The sensitivity of ASL, representing its ability to identify individuals with hypoperfusion correctly, is high at 91.03%, with a 95% confidence interval (CI) ranging from 85.16% to 95.14%. The specificity, indicating the capacity to identify those without hypoperfusion accurately, is reported at 85.98% (95% CI: 77.93% to 91.94%). The *Area Under the Curve* (AUC), which serves as a measure of the overall diagnostic accuracy, is 0.89 with 95% CI from 0.84 to 0.92, thereby suggesting a robust performance of ASL in predicting hypoperfusion. The *Positive Predictive Value* (PPV) is 89.80% (95% CI: 83.73% to 94.18%), reflecting the probability that a positive test result corresponds to actual hypoperfusion, while the *Negative Predictive Value* (NPV) is 87.62% (95% CI: 79.76% to 93.24%), indicating the likelihood that a negative result accurately rules out hypoperfusion. The overall diagnostic accuracy of ASL stands at 88.89%, thus proving its efficacy in assessing hypoperfusion in the study population (see Chart 1).

**Table 3:** Sensitivity, specificity, positive predictive value, and negative predictive value of ASL for predicting hypoperfusion in different areas

Variables	Frontal	Occipital	Parietal	Posterior cingulate	Sensorimotor	Temporal
Sensitivity (95% CI)	91.11% (78.78%-97.52%)	66.67% (9.43%-99.16%)	94.12% (83.76%-98.77%)	91.67% (61.52%-99.79%)	100.00% (2.50%-100.00%)	87.88% (71.80%-96.60%)
Specificity (95% CI)	78.57% (49.20%-95.34%)	87.10% (76.15%-94.26%)	71.43% (29.04%-96.33%)	100.00% (2.50% to 100.0%)	100.00% (66.37% to 100%)	85.71% (57.19% to 98.22%)
AUC (95% CI)	0.85 (0.73 to 0.93)	0.77 (0.65 to 0.86)	0.83 (0.71 to 0.91)	0.96 (0.69 to 1.00)	1 (0.69 to 1.00)	0.87 (0.74 to 0.95)
Positive Predictive Value (95% CI)	93.18% (81.34%-98.57%)	20.00% (2.52%-55.61%)	96.00% (86.29%-99.51%)	100.00% (71.5%-100.00%)	100.00% (2.50%-100.00%)	93.55% (78.58%-99.21%)
Negative Predictive Value (95% CI)	73.33% (44.9%-92.21%)	98.18% (90.28%-9.95%)	62.50% (24.49%-91.48%)	50.00% (1.26%-98.74%)	100.00% (66.37%-100.00%)	75.00% (47.62%-92.73%)
Diagnostic accuracy	88.14%	86.15%	91.38%	92.31%	100.00%	87.23%

Table 3 presents the sensitivity, specificity, positive predictive value (PPV), negative predictive value (NPV), and diagnostic accuracy of *Arterial Spin Labeling* (ASL) for predicting hypoperfusion in various brain regions. In the frontal area, ASL demonstrates a high sensitivity of 91.11% (95% CI: 78.78% to 97.52%) and a specificity of 78.57% (95% CI: 49.20% to 95.34%), resulting in a diagnostic accuracy of 88.14%. The occipital region shows a lower sensitivity of 66.67% (95% CI: 9.43% to 99.16%) and a higher specificity of 87.10% (95% CI: 76.15% to 94.26%), yielding an accuracy of 86.15%. In the parietal region, ASL exhibits a robust sensitivity of 94.12% (95% CI: 83.76% to 98.77%) and a specificity of 71.43% (95% CI: 29.04% to 96.33%), resulting in a diagnostic accuracy of 91.38%.

The posterior cingulate area demonstrates exceptional performance with a sensitivity of 91.67% (95% CI: 61.52% to 99.79%), specificity of 100.00% (95% CI: 2.50% to 100.00%), and an AUC of 0.96 (95% CI: 0.69 to 1.00), leading to a diagnostic accuracy of 92.31%. The sensorimotor and temporal regions exhibit perfect sensitivity and specificity, achieving 100% diagnostic accuracy. In the temporal region, ASL exhibits a robust sensitivity of 87.88% (95% CI: 71.80% to 96.60%) and a specificity of 85.71% (95% CI: 57.19% to 98.22%), resulting in a diagnostic accuracy of 87.23%.

ASL’s positive predictive values range from 20.00% to 100.00%, whereas its negative predictive values range from 50.00% to 100.00% across different brain areas. These results highlight the variability in the ASL performance across the brain regions, thus emphasizing its potential for accurate hypoperfusion prediction in specific regions, particularly in the posterior cingulate, sensorimotor, and temporal areas (see Table 3).

**Table 4:** Sensitivity, specificity, positive predictive value, and negative predictive value of ASL for predicting hypoperfusion in different ACE-3 scores

Variables	ACE-3 Score			
	1–25	26–50	51–75	>75
Sensitivity (95% CI)	89.29% (71.77%-97.73%)	88.89% (51.75%-99.72%)	88.10% (74.37%-96.02%)	92.86% (80.52%-98.50%)
Specificity (95% CI)	100.00% (71.51%-100.00%)	62.50% (24.49%-91.48%)	83.33% (67.19%-93.63%)	89.66% (72.65%-97.81%)
AUC (95% CI)	0.95 (0.82 to 0.99)	0.76 (0.49 to 0.93)	0.86 (0.76 to 0.93)	0.91 (0.82 to 0.97)
Positive Predictive Value (95% CI)	100.00% (86.28%-100.00%)	72.73% (39.03%-93.98%)	86.05% (72.07%-94.70%)	92.86% (80.52%-98.50%)
Negative Predictive Value (95% CI)	78.57% (49.20%-95.34%)	83.33% (35.88%-99.58%)	85.71% (69.74%-95.19%)	89.66% (72.65%-97.81%)
Diagnostic accuracy	92.31%	76.47%	85.90%	91.55%

Table 4 provides insights into the sensitivity, specificity, *positive predictive value* (PPV), *negative predictive value* (NPV), and diagnostic accuracy of *Arterial Spin Labelling* (ASL) for predicting hypoperfusion based on different ACE-3 (*Addenbrooke’s Cognitive Examination*) score ranges. In individuals with an ACE-3 score between 1–25, ASL exhibits a high sensitivity of 89.29% (95% CI: 71.77% to 97.73%) and a perfect specificity of 100.00% (95% CI: 71.51% to 100.00%), resulting in an impressive diagnostic accuracy of 92.31%. For ACE-3 scores between 26–50, ASL shows a sensitivity of 88.89% (95% CI: 51.75% to 99.72%) and a specificity of 62.50% (95% CI: 24.49% to 91.48%), yielding a diagnostic accuracy of 76.47%.

Meanwhile, in the ACE-3 score range of 51–75, ASL maintains a high sensitivity of 88.10% (95% CI: 74.37% to 96.02%) and a specificity of 83.33% (95% CI: 67.19% to 93.63%), resulting in a diagnostic accuracy of 85.90%. For individuals with an ACE-3 score greater than 75, ASL demonstrates excellent sensitivity of 92.86% (95% CI: 80.52% to 98.50%) and a specificity of 89.66% (95% CI: 72.65% to 97.81%), leading to a diagnostic accuracy of 91.55%. The positive predictive values range from 72.73% to 100.00%, while the negative predictive values range from 78.57% to 89.66% across different ACE-3 score categories. These findings underscore the potential of ASL to effectively predict hypoperfusion, with its performance varying across distinct cognitive assessment score ranges (see Table 4).

**Table 5:** Receiver operating characteristic curve of ACE-3 score for predicting the concordance of ASL and PET Z score (-)

Variables	Values
Area under the ROC curve (AUC)	0.55
Standard Error	0.0556
95% Confidence interval	0.479 to 0.620
P value	0.366
Cut off	>82
Sensitivity (95% CI)	24.31% (18.3–31.2%)
Specificity (95% CI)	91.67% (73.0–99.0%)
PPV (95% CI)	95.7% (85.2–99.5%)
NPV (95% CI)	13.8% (8.9–20.2%)
Diagnostic accuracy	32.20%

**Chart 2:**
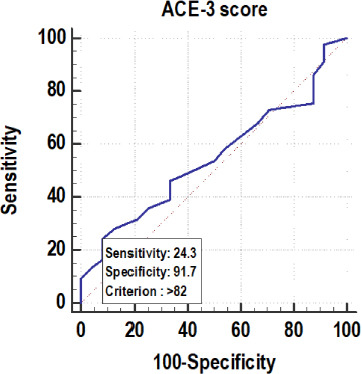
Receiver operating characteristic curve of ACE-3 score for predicting the concordance of ASL and PET Z score (-)

The above-presented Table 5 shows that, among the patients with a concordance of ASL and PET Z score (-), 24.31% had ACE-3 scores>82. If the ACE-3 score is >82, then there is a 95.70% probability of concordance of ASL and the PET Z score (-), whereas, if the ACE-3 score <=82, then there is a 13.80% chance of a discordance of ASL and the PET Z score (-). Among patients with a discordance of ASL and PET Z score (-), 91.67% had an ACE-3 score <=82 (cf. Table 5, Chart 2).

The mean ACE-3 score in the concordant cases was 59.85 ± 26.36, while, in discordant instances, it was 58.46 ± 23.28. There was no significant association between these scores, as indicated by a p-value of 0.806.

## Discussion

A most striking finding was the similarity upon the visual inspection of the patterns of CBF on ASL-MRI and metabolism on FDG-PET. Our study showed that ASL can detect a hypo-perfusion region in the brain with a 91% sensitivity, 85.98% specificity, 89.8% PPV, and 87.62% NPV, with an exemplary overall diagnostic accuracy of 88.9%. These findings are in line with the study by Musiek et al. [19] , which compared the FDG-PET and ASL-MRI findings in patients with clinically diagnosed AD and revealed that both modalities show similar regional abnormalities, as well as a comparable sensitivity and specificity for the detection of AD after a visual review by two expert readers. They proved an excellent diagnostic accuracy for both modalities, with the area under receiver operating characteristic curves of 0.90 for FDG-PET (95% CI: 0.79–0.99) and 0.91 for ASL-MRI (95% CI: 0.80–1.00).

As established by authors of previous researches, there is a correlation between FDG-PET and ASL-MRI in mild-moderate AD patients (19,21,22) and MCI patients (45,46). However, earlier studies in MCI patients had limitations of a smaller sample size, as indicated by Dolui et al. (24), or the absence of temporal proximity in the acquisition of PET and ASL, as given by Tosun et al. (23). We tried to include an equal proportion of patients (37%) with MCI and dementia and found 88% sensitivity, 83% specificity, and 86% diagnostic accuracy in the ASL-PET concordance. This proves that ASL can be a valuable alternative to PET in patients with MCI.

Overall, the literature presents conflicting results – specifically, a metanalysis by Haidar et al. (25) revealed, as per the SROC-curve and AUC measures, that FDG-PET, with an AUC of 86.7%, displays better diagnostic performance than ASL-MRI, with 84.2%. The pooled sensitivity of FDG-PET was 0.858, which is significantly higher than ASL-MRI (0.71). The specificity was somewhat similar among both techniques [0.863 (FDG-PET) versus 0.834 (ASL-MRI)]. The study hinted that ASL-MRI could replace FDG-PET if one can prove a higher sensitivity of ASL than FDG-PET. Our study stands as a continuation of the metanalysis, showing higher sensitivity in the PET-ASL correlation.

We went a step further to analyze the performance of ASL for different brain regions. Our study highlights the higher potential of ASL perfusion prediction in the posterior cingulate, sensorimotor, and temporal areas. These findings are unique, as, to the best of our knowledge, no study in the literature has yet compared the performance of ASL-PET concordance in different brain regions. This is important as deficits in other cognitive domains present differently on imaging. A better concordance in the posterior cingulate and temporal areas means that most dementia patients can have ASL as their primary functional study, which can be later confirmed by a specific biomarker PET, rather than subjecting patients to two radiation studies.

We also compared the performance of ASL for a correct prediction of perfusion at different clinical cognitive scores. Although ASL performed well in all groups of ACE-3 scores, we found better use in the lowest (1–25) and highest ranges (76–100). For the lowest group (1–25), a high sensitivity of 89.29% (95% CI: 71.77% to 97.73%) and a perfect specificity of 100.00% (95% CI: 71.51% to 100.00%) were yielded, resulting in an impressive diagnostic accuracy of 92.31%. For the highest group, an excellent sensitivity of 92.86% (95% CI: 80.52% to 98.50%) and a specificity of 89.66% (95% CI: 72.65% to 97.81%) were obtained, leading to a diagnostic accuracy of 91.55%. This is another research sphere in which we could not find any previous composite study. We found our data robust, as a moderately negative significant correlation was noted between the PET data value range (highest Z Score – lowest Z Score) and the ACE-3 scores. We documented that as the ACE-3 clinical score of the patient increases (i.e., an improvement of cognition), the brain shows a more uniform metabolism with a reduced range of the PET Z Score values. Most of the data in the literature had limitations in the form of a small sample size or an inter-observer variability, but our data, despite being limited, was sufficiently robust for analysis.

A few authors, specifically, Chen et al. [21] and Chételat et al. (26), demonstrated significant discrepancies in the brain atrophy patterns from the hypoperfusion and hypometabolism patterns. It is suggested to be a result of diaschisis, in which functional deficits occur in areas of local neuronal loss and the distant regions of denervation. Our study mostly showed coherence, such that hypoperfusion was noted in the regions of parenchymal atrophy. In conclusion, ASL-MRI measurements of CBF significantly overlap with measures of ^18^F-FDG-PET SUVR in the known regions of early AD neurodegeneration.

## Limitations

We could not conduct the PET and MRI on the same day due to resource constraints. Despite our maximal efforts, approximately 20% of our patients were lost to attrition. We could have included more cases with varying grades of cognition in the study if the study time had been longer. Processing cases, including ROI in ASL, were manual, which incurred a lot of intra-observer variability. No definite correlation with the clinical characteristics (except for an overall cognitive score, like ACE-3), biomarker PET (like amyloid/tau), or histopathology was done for the study. Future work should compare more advanced ASL-MRI methodologies with ^18^F-FDG-PET in the same scanning session in longitudinal studies, where their predictive value for disease progression could be evaluated.

## Conclusion

Despite multiple studies correlating FDG-PET and ASL-MRI in different grades of clinical cognition, a vast controversy has been observed. Our study extends the previous literature on a positive side. We have demonstrated an exceptional 91% sensitivity, 86% specificity, and a diagnostic accuracy of 89% of the PET-ASL concordance. We have also demonstrated a better PET-ASL concordance for the posterior cingulate, temporal, and sensorimotor regions. The ASL-PET concordance was good enough at all clinical cognitive grades, but better at the lowest (1–25 ACE-3 Score) and highest (76–100) cognitive scores.

## Abbreviations

ACE-3: Addenbrooke’s Clinical Examination

AD: Alzheimer’s Disease

ASL: Arterial Spin Labelling

CBF: Cerebral Blood Flow

DALY: Disability-Adjusted Life Years

DLB: Dementia with Lewy Bodies

FDG: Fluorodeoxy Glucose

FTLD: Fronto-Temporal Lobar Dementia

FUS: Fused in Sarcoma

MCI: Mild Cognitive Impairment

MMSE: Mini-Mental State Examination

MTA: Medial Temporal Atrophy

MTI: Magnetization Transfer Imaging

NFT: Neurofibrillary Tangles

WHO: World Health Organization
